# A novel pleuromutilin antibacterial compound, its binding mode and selectivity mechanism

**DOI:** 10.1038/srep39004

**Published:** 2016-12-13

**Authors:** Zohar Eyal, Donna Matzov, Miri Krupkin, Susanne Paukner, Rosemarie Riedl, Haim Rozenberg, Ella Zimmerman, Anat Bashan, Ada Yonath

**Affiliations:** 1 Department of Structural Biology, Weizmann Institute of Science, Rehovot 7610001, Israel; 2 Nabriva Therapeutics AG, 1110 Vienna, Austria

## Abstract

The increasing appearance of pathogenic bacteria with antibiotic resistance is a global threat. Consequently, clinically available potent antibiotics that are active against multidrug resistant pathogens are becoming exceedingly scarce. Ribosomes are a main target for antibiotics, and hence are an objective for novel drug development. Lefamulin, a semi-synthetic pleuromutilin compound highly active against multi-resistant pathogens, is a promising antibiotic currently in phase III trials for the treatment of community-acquired bacterial pneumonia in adults. The crystal structure of the *Staphylococcus aureus* large ribosomal subunit in complex with lefamulin reveals its protein synthesis inhibition mechanism and the rationale for its potency. In addition, analysis of the bacterial and eukaryotes ribosome structures around the pleuromutilin binding pocket has elucidated the key for the drug’s selectivity.

*Staphylococcus aureus* is one of the leading causes of hospital and community associated infections such as skin and soft-tissue infections, sepsis, pneumonia and toxic shock syndrome^1^,^2^. These infections are treated by a variety of antibiotic classes including those targeting the large ribosomal subunit such as oxazolidinones, pleuromutilins, macrolides, lincosamides and ketolides. *S. aureus* possess 5–6 copies of ribosomal RNA (rRNA), operons, and hence acquisition of single nucleotide mutations in the 23S rRNA, which are among the common resistance mechanisms with respect to ribosomal active antibiotics, should have appeared after relatively long periods^3^. Nevertheless the epidemiology of *S. aureus* is increasing^1^.

Among other mechanisms, resistance in *S. aureus* is associated with post-transcription modifications in rRNA and mutations in ribosomal proteins (rProtein). In many cases methicillin-resistant *Staphylococcus aureus* (MRSA) is also resistant to other antibacterial agents such as aminoglycosides, macrolides, lincosamides, tetracyclines, cephalosporins, carbapenems, beta-lactamase inhibitor combinations, trimethoprim and sulphonamides^4^. The prevalence of MRSA strains has created an urgent demand for novel therapeutic agents effective against it.

The pleuromutilins, discovered as *S. aureus* inhibitors in 1951, are produced by the genus *Pleurotus*^5^. These natural products consist of a tricyclic mutilin core, a C21 keto group and various C14 extensions. It was shown that pleuromutilin derivatives mediate inhibition of bacterial protein biosynthesis by binding to the peptidyl transferase center (PTC) at the A- and P- sites^6^,^7^,^8^. Tiamulin was the first family member that was approved for veterinary use in 1979, followed by valnemulin in 1999. Retapamulin is a topically administered pleuromutilin that was approved for human use in 2007.

In the crystal structures of various pleuromutilins complexes with the large ribosomal subunit of *Dienococcus radiodurans* (D50S)^9^,^10^, *Haloarcula marismortui (H50S)*^11^ *and Staphylococcus aureus* (SA50S)^12^ the tricyclic mutilin core is blocking the A-site and its 4-amino-2-hydroxy-cyclohexyl-sulfanyl-acetyl C14 extension is pointing into the P-site, thus perturbing tRNA accommodation at the A- and P-site.

Known ribosomal resistance mechanisms to pleuromutilins include post-transcription modification in the 23S ribosomal RNA by the *Cfr* enzyme which methylates the C8 position of the PTC nucleotide A2503 (*E. coli* rRNA numbering is used throughout)^13^. In addition, resistance occurs by mutations in the ribosomal protein (rProtein) uL3 G144-D159 loop and uL4 G69 residue (*S. aureus* rProtein numbering is used throughout)^14^,^15^, which is located in proximity to the PTC.

Lefamulin (also known as BC-3781) developed by Nabriva Therapeutics, Vienna, Austria, is a highly active semi-synthetic pleuromutilin compound against pathogens that are commonly associated with community-acquired bacterial pneumonia (CABP), including multidrug resistant *S. pneumoniae, S. aureus*, and *M. pneumonia*^16^. Lefamulin has been evaluated in a phase II trial, which demonstrated the first proof of concept for the systemic use of a pleuromutilin antibiotic for the treatment of acute bacterial skin and skin structure infection (ABSSSI)^17^. Currently, it is in phase III development for treatment of community-acquired bacterial pneumonia (CABP).

## Results

### Structure of the *S. aureus* 50S in complex with lefamulin

**Figure 1:**
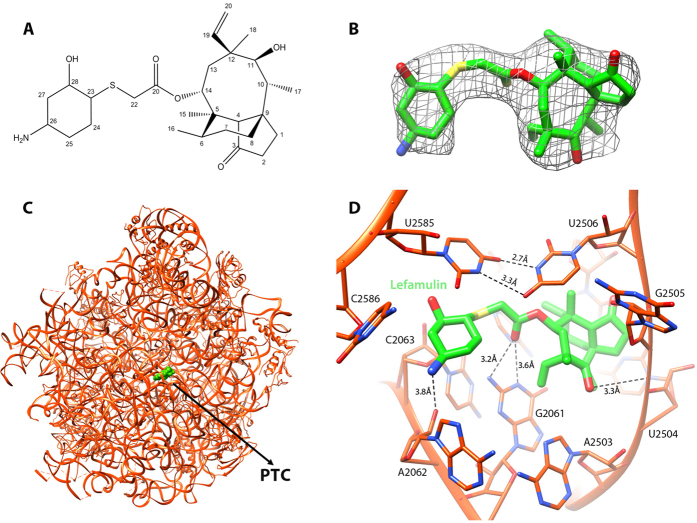
(**A**) The chemical structure of lefamulin. (**B**) The weighted *2F*_*o*_*-F*_*c*_ electron density map of the complex S50S-lefamulin around the lefamulin’s binding site contoured at 1.0σ. **(C)** The binding site of lefamulin (green) within the SA50S-lef structure at the PTC **(D)** Zoom into the binding pocket where lefamulin (green) is held within the PTC 23S rRNA nucleotides (orange) with hydrogen bonds (dashed lines). The U:U interactions between U2585 and U2506 stabilize the lefamulin binding pocket.

We determined the crystal structure of the large ribosomal subunit of *S. aureus* with lefamulin (SA50S-lef) (Table 1), from which we could readily identify the lefamulin molecule. An electron density map around lefamulin is shown in Fig. 1.

**Figure 2:**
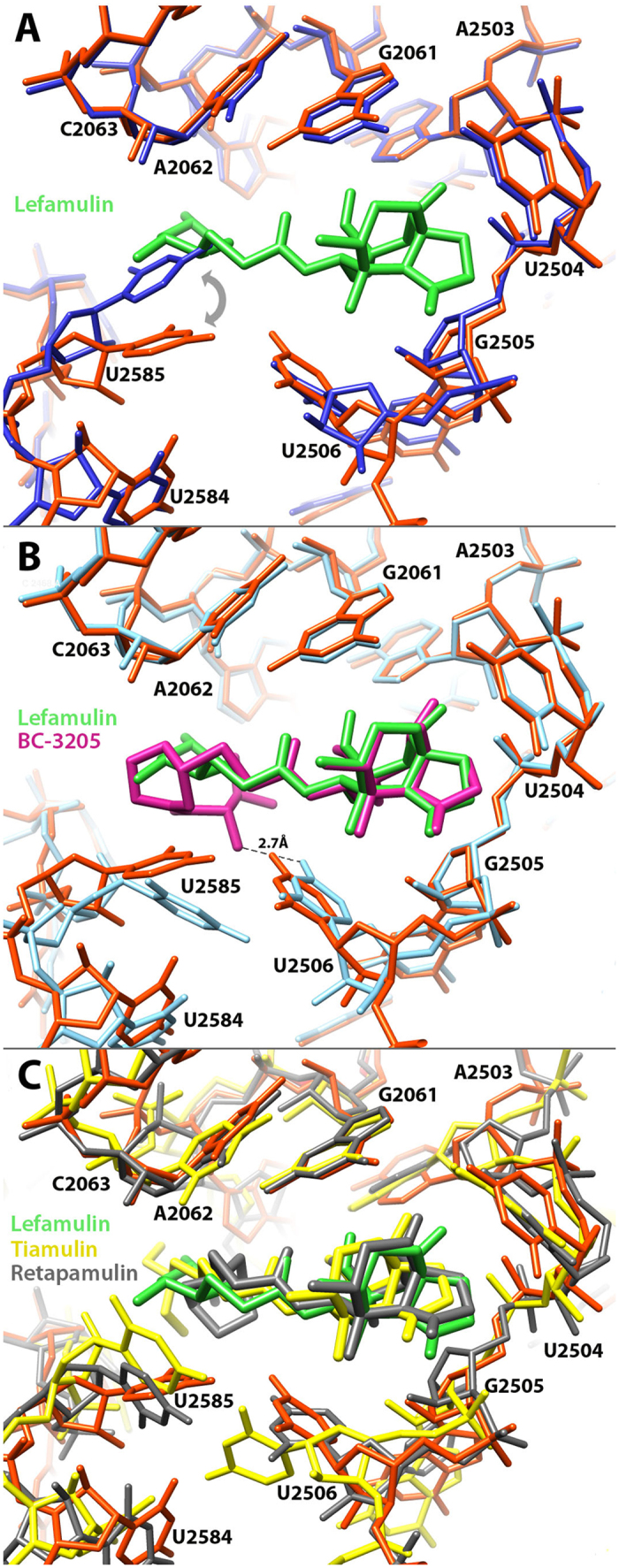
Comparative analysis of the lefamulin binding pocket. (**A**) A superposition of the PTC in apo SA50S structure (blue) and in the SA50S-lef complex (lefamulin in green 23S rRNA in orange) reveals relocations of nucleotides U2585 and U2506 in the bound *vs.* apo structure. (**B**) A superposition of the PTC in the two *S. aureus* pleuromutilins complexes [SA50SBC3205 (teal and pink) (4WFB) and SA50S-lef (orange and green) - this work. The dash line represents the unique hydrogen bond BC-3205 forms with the 23S rRNA. (**C**) A superposition of pleuromutilins within their binding pockets of *S. aureus* and *D. radiodurans* [SA50S-lef (orange and green) - this work, D50S-retapamulin (dark grey) (2OGO)^10^, D50S-tiamulin (yellow) (1XBP)^9^].

In the SA50S-lef crystal structure, lefamulin is bound at the PTC (Fig. 1), which is in agreement with other pleuromutilin complexes of D50S^9^,^10^, H50S^11^ and SA50S^12^ (Fig. 2).

In the available ribosome-pleuromutilin complex structures, all pleuromutilins bind to ribosomes at the same pocket and form three hydrogen bonds between the drug’s acetyl carbonyl with the NH and NH_2_ of G2061 and between C11 hydroxyl group with the phosphate group of G2505 (Fig. 1D). In the SA50S-lef complex, U2585 is stacked with the lefamulin’s C14 extension, which is a non-planar cyclohexane moiety^18^,^19^ (Fig. 1). This interaction is stabilized by a U-U-4-carbonyl N3 symmetric interactions between U2585 and U2506 that, upon binding, are shifted from the binding pocket (Fig. 2A). Interactions between these two nucleotides have been identified previously in several D50S pleuromutilin complexes^10^ (Fig. 2C). A unique hydrogen bond between 23S rRNA nucleotides and lefamulin is formed between NH_2_ group of lefamulin’s C14 extension and the O2 of the A2062 ribose. All of the other interactions of lefamulin with the rRNA nucleotides C2063, U2506, A2503, U2504, G2505, A2453, C2452, A2425 and C2424 are either hydrophobic or based on Van der Waals forces.

In the SA50S-lef complex structure, the conformation adopted by the flexible nucleotide U2585 is different from that in the structures: apo-SA50S, SA50S-BC3205^12^, H50S-tiamulin^11^ and D50S-pleuromutilin^9^,^10^ (Fig. 2).

**Figure 3:**
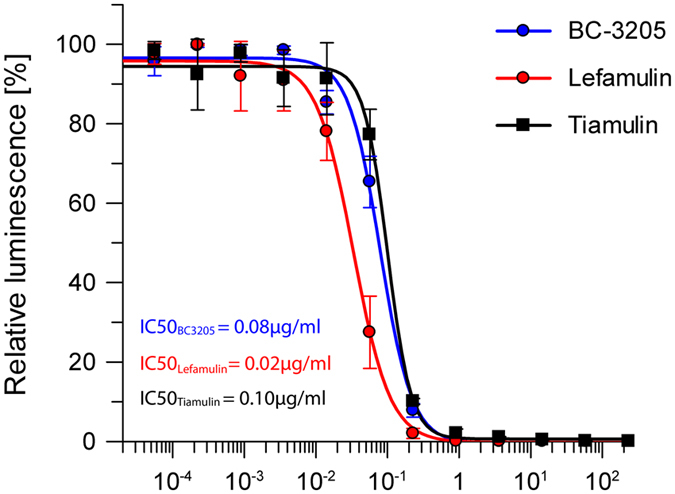
Inhibition of bacterial protein synthesis. The inhibitory effect of BC-3205, lefamulin and tiamulin on protein expression in *S. aureus in vitro* transcription-translation cell-free system. The activity of the reporter protein (luciferase) in the presence of various concentrations of BC-3205, lefamulin and tiamulin is shown as arbitrary unit of luminescence [a.u.]. The IC_50_ values calculated by the plotted data showed better inhibition of protein synthesis by lefamulin than by BC-3205 and tiamulin.

Lefamulin seems to make the same number of hydrogen bonds with the PTC nucleotides as BC-3205. Stabilization of lefamulin in its binding pocket is achieved by U2585 and U2506 U:U interactions. This agrees with the IC_50_ values that were determined in *S. aureus* cell free *in vitro* transcription-translation assay for BC-3205, lefamulin and tiamulin: (IC_50_ = 0.02 μg/ml of lefamulin, IC_50_ = 0.08 μg/ml of BC-3205, IC_50_ = 0.10 μg/ml of tiamulin (Fig. 3) Moreover, inhibition of *in vitro* transcription-translation show lower IC_50_ values for lefamulin than for other known pleuromutilins acting on *S. aureus*^20^.

### Structural basis for pleuromutilins selectivity

Selectivity, namely the distinction between bacterial pathogens and eukaryotes, is crucial for the clinical use of antibiotics. Ribosome inhibiting antibiotics, which are currently in clinical use, target bacterial ribosomes without hampering the activity of the eukaryote’s ribosome.

By comparing the SA50S structure with archaeal and eukaryotic ribosome structures, namely those of *Haloarcula marismortui* 50S^11^*, Saccharomyces cerevisiae* 80S^21^, *Tetrahymena thermophilia* 60S^22^, *Leishmania donovani* 60S^23^ and human (*Homo sapiens*) 80S^24^ the previously suggested mechanism by which A-site cleft antibiotic selectivity could be extended^11^,^25^.

**Figure 4:**
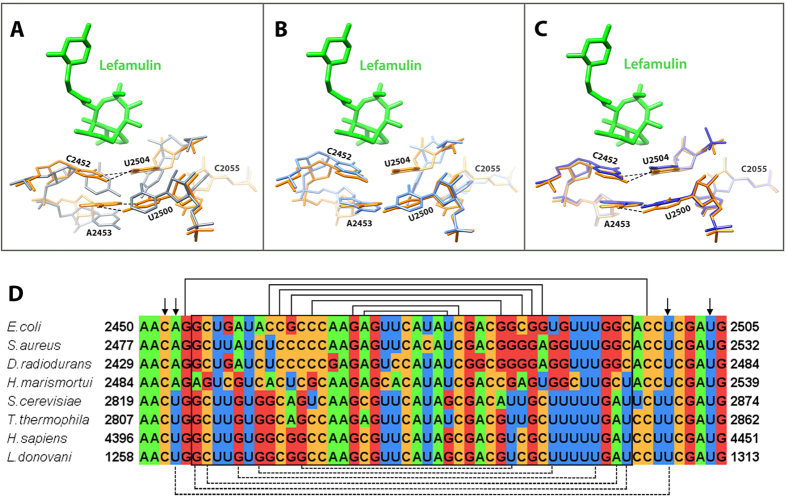
The binding pocket of lefamulin. (**A**) SA50S-lef (lefamulin in green and 23S rRNA in orange) superimposed on the structure of human 80S (grey) (4UG0). In *S. aureus* nucleotides C2452 and U2504 interact with each other (marked with dashed lines) while the same nucleotides in eukaryotes’ structures have the same identity but different orientations, so they cannot interact with each other. Nucleotides A2543 and U2500 are paired (marked with dashed lines) and stacked to C2452 and U2504 in the bacterial ribosomes. In eukaryotes’ structures, nucleotides 2453 and 2500 are both uridines and no interaction between them occurs owing to their orientation. In eukaryotes, U2504 has different orientation than in bacteria which allows pi stacking to A2055 (C in bacteria, A in eukaryotes). This interaction stabilizes the “open” conformation of the PTC which is the key for the pleuromutilins’ selectivity. (**B**) A superposition of the rRNA H50S-tiamulin structure (light blue) (3G4S) on that of SA50S-lef (orange). In archaea, nucleotides A2543 and U2500 cannot form Watson-Crick base-pair due to the stacking between U2504 and A2055. (**C**) A superposition of the rRNA apo SA50S structure (blue) (4WCE) on the SA50S-lef (orange) shows that the position of these nucleotides is the same in the bound and the unbound 50S. (**D**) Multiple sequence alignment of part of the pleuromutilin binding site, comparing the bacterial, archaeal and eukaryotes large ribosomal subunit rRNA. Helix 89 nucleotides, which are part of the PTC, are marked in a box. Arrows are indicating on the nucleotides C2452, A/U2453, U2504 and U2500 which are 1^st^ and 2^nd^ shell from the mutilin moiety. The lines represent Watson-crick base pairs, dashed lines are base pairs only in bacteria.

As already suggested, in all known bacterial ribosome with pleuromutilins, nucleotides C2452 and U2504 are part of the pleuromutilin binding pocket. Nucleotides U2504 and C2452 hydrophobically interact with the tricyclic mutilin core. These nucleotides interact with each other by non-Watson-Crick C:U 4-carbonyl-amino N3-N3 interactions^26^. In contrast, the respective nucleotides in eukaryotic and archaeal ribosomes of the same sequence identity are positioned in different orientations thus cannot interact with each other (Fig. 4A). As described previously, in archaeal and eukaryotic ribosomes, nucleotides U2504 forms pi stacking interaction to A2055 (in archaea and eukaryotes) that is pointing away from the PTC binding site (Fig. 4A and B). This interaction pushes U2504 out of the binding pocket that opens up, thus pleuromutilins binding is hampered.

Additional observations indicate that for bacterial ribosome, the PTC 2^nd^ shell base-pair A2543-U2500, is an important determinant for the bacterial drug binding. This base-pair keeps the pleuromutilin binding pocket in a “closed” form by stacking to C2452 and U2504 which interact with the mutilin core. In contrast, in the eukaryotic ribosome, both nucleotides 2453 and 2500 are uridines, and do not interact with each other (Fig. 4A and B), thus an “open” conformations is achieved. This additional base-pair at the 2^nd^ shell from the pleuromutilin may contribute extra stability to the binding pocket and may play an important role in their selectivity mechanism. In archaea, a Watson-Crick base-pair formation of nucleotides A2543 and U2500 is hindered due to the stacking between U2504 and A2055. Consequently, the complete selectivity mechanism could not be rationalized by archaeal ribosome-tiamulin structure. Indeed, the archaeal ribosome is not sensitive to tiamulin. Hence the drug concentration that was needed in order to create the H50S-tiamulin complex was 100 times higher than the concentration for creating the tiamulin-D50S complex^27^.

The double layer nucleotides around the pleuromutilin tricyclic mutilin core create a chemically and structurally different binding pocket in prokaryotes vs. eukaryotes and archaea. The tricyclic mutilin core, which is the common moiety for all the pleuromutilins, is used as an anchor for the drug binding. Although the tricyclic motilin core doesn’t form any hydrogen bonds with the PTC nucleotides, it is stabilized by hydrophobic and Van der Waals interactions. Compared to the pocket part that binds the mutilin core in eukaryotes and archaea, in eubacteria this part of the pocket is more rigid and chemically stabilized by the C:U interaction in the 1^st^ shell stacked to the A:U base pair in the 2^nd^ shell. Also, since in eukaryotes and archaea, the walls of the binding pocket are open, the tricyclic moiety cannot be properly anchored and consequently no binding or inhibition by pleuromutilins can be stabilized. In this way selectivity towards prokaryotes is achieved.

## Discussion

The binding of lefamulin to the *S. aureus* large ribosomal subunit seems to be tighter than those produced by other pleuromutilins. Its binding triggers an induced fit rearrangement similar to that observed for other pleuromutilins, but in SA50S-lef complex an additional specific U:U interaction is formed. This interaction serves as a physical barrier that maintains a rather tight binding pocket conformation and might be the reason for the lefamulin higher potency over BC-3205 in *S. aureus* cell free *in vitro* transcription-translation assay.

Even though the PTC rRNA nucleotides are conserved across all kingdoms of life, the pleuromutilin family targets selectively bacterial ribosome, without inhibiting eukaryotic ribosomes. This selectivity originates from sequence differences. In Eukaryotic ribosome, the 2^nd^ shell nucleotides are arranged such that they induce a structurally different conformation for the tricyclic mutilin binding site, namely a more “open” conformation, hence hampering the pleuromutilins binding and facilitating their clinical usage. Our current and previous studies revealed the factors governing the inhibition and highlighted additional aspects of pleuromutilins selectivity, and thus may extend the design of additional derivatives of these potent antibacterial drugs for the treatment against mutli-drug resistant bacterial strains.

## Materials and Methods

### *S. aureus* growth and cell wall disruption

*S. aureus* strain RN4220 (American Type Culture Collection 35556)^28^ was grown and disrupted as described previously^12^.

### Ribosome Purification, Crystallization, and Compound Soaking Experiments

Ribosomes were purified as described previously^12^. SA50S was crystallized at 20 °C by the hanging-drop vapor diffusion technique. The crystallization drop contained 0.166% MPD, 0.333% EtOH, 20 mM Hepes pH range 6.8–7.8, 10 mM MgCl_2_, 60 mM NH_4_Cl, 15 mM KCl, 5 mM spermidine, 0.5 mM MnCl2, and 1–1.6 mg/mL SA50S subunits. The reservoir solution contained 15% of 1:2 ethanol-MPD and 110 mM Hepes pH range 6.8–7.8, 10 mM MgCl_2_, 60 mM NH_4_Cl, 15 mM KCl (pH range, 6.8–7.8) as previously described^12^. For obtaining SA50S-lefamulin complex, SA50S crystals were soaked in stabilization solutions containing 44.9 μM (22.8 μg/ml) lefamulin in the stabilization solution for 6 hrs prior to flash freezing and data collection.

### Data collection and processing

Before data collection the crystals were immersed in cryoprotectant solution of 20% MPD, 15% EtOH, 110 mM Hepes pH range 6.8–7.8, 10 mM MgCl_2_, 60 mM NH_4_Cl, 15 mM KCl and 0.5 mM MnCl_2_. Crystallographic data were collected at the ID23-1 beamlines, at the European Synchrotron Radiation Facility (ESRF), Grenoble, France. X-ray diffraction data were collected from the hexagonal crystals at 100 K. Up to 15 crystals were needed for yielding complete datasets of SA50S using 0.1° oscillations. Data were processed with HKL2000^29^ and CCP4 package suite^30^.

### Map calculation, model building and refinement

The apo-SA50S structure (PDB code: 4WCE) was used as a starting model for rigid body and positional refinement as implemented in PHENIX^31^. Densities for the antibiotics were located using a standard difference *Fourier* maps. For R-free calculations during refinement cycles, random 5% of the data were omitted during refinement cycles. Modelling of the ribosomal RNA and the ribosomal proteins according to the electron density maps was performed using Coot^32^,^33^, Rosetta ERRASER^34^ was used to facilitate further building and to improve the quality of the rRNA geometry. Figures were generated using Pymol^35^ and Chimera^36^. Sequence alignments were performed using BLAST^37^ Structure alignments were done using LSQMAN^38^ and Coot.

### Sequence alignment

Sequence alignment was done with MAFFT version 7^39^ and its figure was done by Jalview software^40^.

### Ribosome Inhibition assay

The inhibition effect of BC-3205 and lefamulin on *S. aureus* ribosomes was tested in a bacterial coupled transcription/translation assay system, in the presence of increasing concentrations of the compounds, which measures the expression of the luciferase gene^41^. The luciferase gene was inserted into plasmid with T7 RNA polymerase promoter. The reaction mixture contained: 160 mM Hepes-KOH (pH 7.5), 6.5% PEG 8 K, 0.074 mg/ml tyrosine, 1.3 mM ATP, 0.86 mM CTP, GTP and UTP, 208 mM potassium glutamate, 83 mM creatine phosphate, 28 mM NH_4_OAc, 0.663 mM cAMP, 1.8 mM DTT, 0.036 mg/ml folinic acid, 0.174 mg/ml *E. coli* tRNA mix, 1 mM amino acid, 0.25 mg/ml creatine kinase, 0.027 mg/ml T7 RNA polymerase, ribosome free *E. coli* cell free extract, 300 nM of *S. aureus* ribosomes, 0.003 μg/μl luciferase plasmid and pleuromutilin compound diluted to 0.46 mM to 0.98 μM (233 μg/ml to 0.05 ng/ml). The reaction mixture was incubated at 37 °C for 1 hr and terminated by the addition of erythromycin at a final concentration of 8 μM. To quantify the reaction’s products, Luciferin Assay Reagent (LAR, Promega) at 5:3 (luciferase: reaction mix) volume ratio was added to the mixture and luminescence was measured. The results were plotted and IC_50_ values were calculated with the program GraFit 7^42^.

### Data deposition

The atomic coordinates and structure factors have been deposited in the Protein Data Bank, www.rcsb.org (PDB ID code: 5HL7).

## Additional Information

**How to cite this article**: Eyal, Z. *et al*. A novel pleuromutilin antibacterial compound, its binding mode and selectivity mechanism. *Sci. Rep.* **6**, 39004; doi: 10.1038/srep39004 (2016).

**Publisher’s note:** Springer Nature remains neutral with regard to jurisdictional claims in published maps and institutional affiliations.

## Figures and Tables

**Table 1:** Crystallographic data and refinement statistics of *S. aureus* 50S in complex with lefamulin (SA50S-lef).

	SA50S-lef
**Crystal information**	
Space group	P6_5_22
a = b [Å]	282.1
c [Å]	875.3
α, β, γ [°]	90, 90, 120
**Diffraction data statistics**	
X-ray source (ESRF)	ID23-1
Wavelength [Å]	0.972
Number of crystals	12
Resolution [Å]	50–3.55 (3.61–3.55)
Observed reflections	2606786
Unique reflections	236087
Redundancy	11.0 (9.2)
Completeness [%]	95.7 (94.6)
<I>/<σ (I)>	9.0 (1.71)
R-merge [%]	20.9 (99.4)
**Refinement statistics**	
R-factor [%]	18.69
R-free (5%) [%]	22.66
rmsd bonds [Å]	0.01
rmsd angles [°]	1.61

*In parentheses are the values for the highest resolution shells. Abbreviations: ESRF, European Synchrotron Radiation Facility; rmsd, root-mean-square deviation.

## References

[b1] Tong S. Y., Davis J. S., Eichenberger E., Holland T. L. & Fowler V. G. Staphylococcus aureus infections: epidemiology, pathophysiology, clinical manifestations, and management. Clinical microbiology reviews 28, 603–661 (2015). 26016486 10.1128/CMR.00134-14 PMC4451395

[b2] Kurosu M., Siricilla S. & Mitachi K. Advances in MRSA drug discovery: where are we and where do we need to be? Expert opinion on drug discovery 8, 1095–1116 (2013). 23829425 10.1517/17460441.2013.807246 PMC3750092

[b3] Besier S., Ludwig A., Zander J., Brade V. & Wichelhaus T. A. Linezolid resistance in Staphylococcus aureus: gene dosage effect, stability, fitness costs, and cross-resistances. Antimicrobial agents and chemotherapy 52, 1570–1572 (2008). 18212098 10.1128/AAC.01098-07 PMC2292563

[b4] Baquero F. Gram-positive resistance: challenge for the development of new antibiotics. Journal of Antimicrobial Chemotherapy 39, 1–6 (1997). 10.1093/jac/39.suppl_1.1 9511055

[b5] Kavanagh F., Hervey A. & Robbins W. J. Antibiotic Substances From Basidiomycetes: VIII. Pleurotus Multilus (Fr.) Sacc. and Pleurotus Passeckerianus Pilat*. Proceedings of the National Academy of Sciences of the United States of America 37, 570 (1951). 16589015 10.1073/pnas.37.9.570 PMC1063423

[b6] Hodgin L. A. & HÖGenauer G. The Mode of Action of Pleuromutilin Derivatives. European Journal of Biochemistry 47, 527–533, doi: 10.1111/j.1432-1033.1974.tb03721.x (1974). 4611767

[b7] HÖGENAUER G. The mode of action of pleuromutilin derivatives. European journal of Biochemistry 52, 93–98 (1975). 1100373 10.1111/j.1432-1033.1975.tb03976.x

[b8] Poulsen S. M., Karlsson M., Johansson L. B. & Vester B. The pleuromutilin drugs tiamulin and valnemulin bind to the RNA at the peptidyl transferase centre on the ribosome. Molecular microbiology 41, 1091–1099 (2001). 11555289 10.1046/j.1365-2958.2001.02595.x

[b9] Schlünzen F., Pyetan E., Fucini P., Yonath A. & Harms J. r. M. Inhibition of peptide bond formation by pleuromutilins: the structure of the 50S ribosomal subunit from Deinococcus radiodurans in complex with tiamulin. Molecular microbiology 54, 1287–1294 (2004). 15554968 10.1111/j.1365-2958.2004.04346.x

[b10] Davidovich C. . Induced-fit tightens pleuromutilins binding to ribosomes and remote interactions enable their selectivity. Proceedings of the National Academy of Sciences 104, 4291–4296 (2007). 10.1073/pnas.0700041104 PMC1817833 17360517

[b11] Gürel G., Blaha G., Moore P. B. & Steitz T. A. U2504 Determines the Species Specificity of the A-Site Cleft Antibiotics: The Structures of Tiamulin, Homoharringtonine, and Bruceantin Bound to the Ribosome. Journal of molecular biology 389, 146–156 (2009). 19362093 10.1016/j.jmb.2009.04.005 PMC2682339

[b12] Eyal Z. . Structural insights into species-specific features of the ribosome from the pathogen Staphylococcus aureus. Proceedings of the National Academy of Sciences 112, E5805–E5814 (2015). 10.1073/pnas.1517952112 PMC4629319 26464510

[b13] Long K. S., Poehlsgaard J., Kehrenberg C., Schwarz S. & Vester B. The Cfr rRNA methyltransferase confers resistance to phenicols, lincosamides, oxazolidinones, pleuromutilins, and streptogramin A antibiotics. Antimicrobial agents and chemotherapy 50, 2500–2505 (2006). 16801432 10.1128/AAC.00131-06 PMC1489768

[b14] Gentry D. R., Rittenhouse S. F., McCloskey L. & Holmes D. J. Stepwise exposure of Staphylococcus aureus to pleuromutilins is associated with stepwise acquisition of mutations in rplC and minimally affects susceptibility to retapamulin. Antimicrobial agents and chemotherapy 51, 2048–2052 (2007). 17404009 10.1128/AAC.01066-06 PMC1891380

[b15] Paukner S., Clark C., Ivezic-Schoenfeld Z. & Kosowska-Shick K. Single- and Multistep Resistance Selection with the Pleuromutilin Antibiotic BC-3781. Fifty-second Interscience Conference on Antimicrobial Agents and Chemotherapy, San Francisco, CA. Poster C1-1971 (2012).

[b16] Paukner S., Sader H. S., Ivezic-Schoenfeld Z. & Jones R. N. Antimicrobial activity of the pleuromutilin antibiotic BC-3781 against bacterial pathogens isolated in the SENTRY Antimicrobial Surveillance Program (2010). Antimicrobial agents and chemotherapy AAC. 00358–00313 (2013). 10.1128/AAC.00358-13 PMC3754340 23836172

[b17] Prince W. . A Phase II clinical study of BC-3781, a pleuromutilin antibiotic, in the treatment of patients with acute bacterial skin and skin structure infection. Antimicrobial agents and chemotherapy AAC. 02106–02112 (2013). 10.1128/AAC.02106-12 PMC3632892 23422913

[b18] François B. . Crystal structures of complexes between aminoglycosides and decoding A site oligonucleotides: role of the number of rings and positive charges in the specific binding leading to miscoding. Nucleic acids research 33, 5677–5690 (2005). 16214802 10.1093/nar/gki862 PMC1251667

[b19] Kondo J., Koganei M. & Kasahara T. Crystal structure and specific binding mode of sisomicin to the bacterial ribosomal decoding site. ACS medicinal chemistry letters 3, 741–744 (2012). 24900542 10.1021/ml300145y PMC4025859

[b20] Paukner S., Strickmann D. B. & Ivezic-Schoenfeld Z. Extended Spectrum Pleuromutilins: Mode-of-Action Studies *24th ECCMID (European Congress of Clinical Microbiology and Infectious Diseases), Barcelona, Spain* (2014).

[b21] Ben-Shem A. . The structure of the eukaryotic ribosome at 3.0 ֳ… resolution. Science 334, 1524–1529 (2011). 22096102 10.1126/science.1212642

[b22] Klinge S., Voigts-Hoffmann F., Leibundgut M. & Ban N. Atomic structures of the eukaryotic ribosome. Trends in biochemical sciences 37, 189–198 (2012). 22436288 10.1016/j.tibs.2012.02.007

[b23] Shalev-Benami M. . 2.8-Å Cryo-EM Structure of the Large Ribosomal Subunit from the Eukaryotic Parasite Leishmania. Cell Reports (2016). 10.1016/j.celrep.2016.06.014 PMC5835689 27373148

[b24] Khatter H., Myasnikov A. G., Natchiar S. K. & Klaholz B. P. Structure of the human 80S ribosome. Nature 520, 640–645 (2015). 25901680 10.1038/nature14427

[b25] de Loubresse N. G. . Structural basis for the inhibition of the eukaryotic ribosome. Nature 513, 517–522 (2014). 25209664 10.1038/nature13737

[b26] Dirheimer G., K. G., Dumas P. & Westhof E. tRNA: Structure, Biosynthesis, and Function (Söll D., RajBhandary U. Eds). (American Society for Microbiology, Washington, 1995).

[b27] Wilson D. N. On the specificity of antibiotics targeting the large ribosomal subunit. Annals of the New York Academy of Sciences 1241, 1–16 (2011). 22191523 10.1111/j.1749-6632.2011.06192.x

[b28] Novick R. P. [27] Genetic systems in Staphylococci. Methods in enzymology 204, 587–636 (1991). 1658572 10.1016/0076-6879(91)04029-n

[b29] Otwinowski Z. & Minor W. Processing of X-ray diffraction data. Methods enzymol 276, 307–326 (1997). 10.1016/S0076-6879(97)76066-X 27754618

[b30] Winn M. D. . Overview of the CCP4 suite and current developments. Acta Crystallographica Section D: Biological Crystallography 67, 235–242 (2011). 21460441 10.1107/S0907444910045749 PMC3069738

[b31] Adams P. D. . PHENIX: a comprehensive Python-based system for macromolecular structure solution. Acta Crystallogr D Biol Crystallogr 66, 213–221 (2010). 20124702 10.1107/S0907444909052925 PMC2815670

[b32] Emsley P. & Cowtan K. Coot: model-building tools for molecular graphics. Acta Crystallogr D Biol Crystallogr 60, 2126–2132 (2004). 15572765 10.1107/S0907444904019158

[b33] Emsley P., Lohkamp B., Scott W. G. & Cowtan K. Features and development of Coot. Acta Crystallogr D Biol Crystallogr 66, 486–501 (2010). 20383002 10.1107/S0907444910007493 PMC2852313

[b34] Chou F.-C., Sripakdeevong P., Dibrov S. M., Hermann T. & Das R. Correcting pervasive errors in RNA crystallography through enumerative structure prediction. Nature methods 10, 74–76 (2012). 23202432 10.1038/nmeth.2262 PMC3531565

[b35] Schrodinger L. L. C. The PyMOL Molecular Graphics System, Version 1.3r1 (2010).

[b36] Pettersen E. F. . UCSF Chimera—a visualization system for exploratory research and analysis. Journal of computational chemistry 25, 1605–1612 (2004). 15264254 10.1002/jcc.20084

[b37] Altschul S. F., Gish W., Miller W., Myers E. W. & Lipman D. J. Basic local alignment search tool. J Mol Biol 215, 403–410 (1990). 2231712 10.1016/S0022-2836(05)80360-2

[b38] Kleywegt G. J. & Jones T. A. Where freedom is given, liberties are taken. Structure 3, 535–540 (1995). 8590014 10.1016/s0969-2126(01)00187-3

[b39] Katoh K. & Standley D. M. MAFFT multiple sequence alignment software version 7: improvements in performance and usability. Molecular biology and evolution 30, 772–780 (2013). 23329690 10.1093/molbev/mst010 PMC3603318

[b40] Waterhouse A. M., Procter J. B., Martin D. M., Clamp M. & Barton G. J. Jalview Version 2—a multiple sequence alignment editor and analysis workbench. Bioinformatics 25, 1189–1191 (2009). 19151095 10.1093/bioinformatics/btp033 PMC2672624

[b41] Murray R. W., Melchior E. P., Hagadorn J. C. & Marotti K. R. Staphylococcus aureus cell extract transcription-translation assay: firefly luciferase reporter system for evaluating protein translation inhibitors. Antimicrobial agents and chemotherapy 45, 1900–1904 (2001). 11353649 10.1128/AAC.45.6.1900-1904.2001 PMC90569

[b42] Leatherbarrow R. J. GraFit Version 7, Erithacus Software Ltd.: Horley, U.K., (2009).

